# Caution in Interpreting the Absence of Appropriate ICD Therapies in Pediatric Primary Prevention

**DOI:** 10.1002/joa3.70332

**Published:** 2026-04-08

**Authors:** Sami Ullah Khan, Syed Huzaifa Khan, Muhammad Faizan Khan

**Affiliations:** ^1^ Nowshera Medical College Nowshera Pakistan; ^2^ Khyber Medical University Peshawar Pakistan

To the Editor,

We read with interest the article by Bjeloševič et al. [1], describing a 14‐year nationwide experience with implantable cardioverter‐defibrillator (ICD) implantation in pediatric patients. The authors supply valuable data on indications, complications, and outcomes in a relatively underrepresented European setting. However, we wish to raise several methodological considerations that merit further discussion.

First, caution is warranted when interpreting the absence of appropriate ICD shocks in the primary‐prevention subgroup. While the study suggests that the lack of observed therapies may reflect uncertainty regarding the benefit of ICD implantation for primary prevention, it is also notable that the primary prevention group included only 10 patients with relatively limited follow‐up. Such small cohorts may be underpowered to reliably detect ventricular arrhythmias, which are clinically important but often occur at low absolute rates among pediatric populations. As a result, the absence of observed shocks might reflect constraints on sample size and follow‐up duration rather than provide evidence against the effectiveness of primary‐prevention ICD therapy. Previous registry analyses of pediatric ICD recipients have demonstrated that appropriate device therapies occur at relatively low rates and are often captured only with larger populations and longer observation periods [2, 3].

Second, the substantial heterogeneity of underlying cardiac conditions within the cohort may further limit the understandability of the reported outcomes. The study population included patients with diverse diagnoses such as long QT syndrome, hypertrophic cardiomyopathy, catecholaminergic polymorphic ventricular tachycardia, and congenital heart disease, each linked with markedly different arrhythmic risks and clinical trajectories. Pooled analysis without disease‐specific stratification may therefore substantially confound the observed incidence of appropriate ICD therapies. Contemporary investigations have emphasized the importance of disease‐specific analyses in pediatric ICD populations, particularly as evolving device programming strategies continue to influence shock incidence [4, 5].

In brief, this nationwide cohort yields valuable data collected on pediatric ICD implantation. However, the absence of appropriate therapies in the primary‐prevention subgroup should be interpreted cautiously, given the limited sample size and follow‐up duration, while the heterogeneous disease composition of the cohort may independently confound outcome estimates. These considerations accentuate the importance of adequately powered, disease‐specific analyses when evaluating the effectiveness of ICD therapy in pediatric primary prevention.

## Author Contributions

All authors have read and approved the final manuscript.

## Funding

The authors have nothing to report.

## Disclosure

Transparency statement: The corresponding author (Sami Ullah Khan) affirms that this manuscript is an honest, accurate, and transparent account of the study reported; that no important aspects of the study have been omitted; and that any deviations from the study as planned have been explained.

## Ethics Statement

The authors have nothing to report.

## Consent

The authors have nothing to report.

## Conflicts of Interest

The authors declare no conflicts of interest.

## Data Availability

No new data were generated in this work.
